# My patient is allergic to eggs, can i use propofol? A case report and review

**DOI:** 10.4103/1658-354X.71581

**Published:** 2010

**Authors:** Jamal Tashkandi

**Affiliations:** *Anaesthesia Consultant, College of Medicine, King Khalid University, Saudi Arabia*

**Keywords:** *Allergy*, *egg allergic patient*, *propofol*

## Abstract

Rather than other drugs, propofol is more likely to be used for induction of anesthesia to cause an allergic reaction. Propofol is becoming the most common intravenous agent used for induction as well as maintenance of anaesthesia. Allergy to propofol is rarely reported. We present a case of 4–year-old boy presented for elective adenotonsillectomy with past medical history of eczema and multiple allergies to food. He developed what seems to be an allergic reaction to propofol. We concluded that anaesthetists should be alerted when using propofol in patients with history of atopy or several drug allergies. Current evidence suggests that egg allergic patients are not more likely to develop anaphylaxis when exposed to propofol. If reactions to drugs occurred, it is always advisable to ascertain the exact allergen in each individual case before deciding causality. Serum tryptase, skin prick, intradermal testing, or serologic testing should be done to confirm the diagnosis of an anaphylactic reaction.

## INTRODUCTION

**Figure 1:**
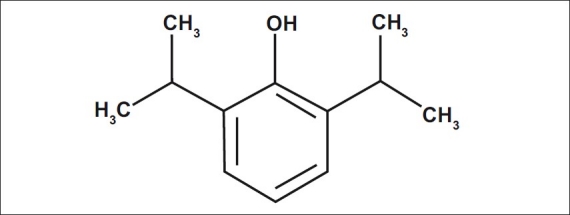
Propofol molecule

Propofol is an intravenous (i.v.) anaesthetic agent, its chemical name is 2,6-di-isopropyl-phenol, C12H18O, it is formulated in an emulsion with intralipid, it contains Propofol 1%w/v, Soya bean oil 10%, glycerol 2.25%, and purified egg phosphatide 1.2% [Figure 1].[1]

Propofol is more likely than other drugs used for induction of anesthesia to cause an allergic reaction, and in one report it was found that 1.2% of cases of perioperative anaphylactic shock were attributable to propofol.[2] The incidence of allergy secondary to propofol has been reported to be 1:15 000 anaesthetics, irrespective of the mechanism involved. The largest report of allergic reactions to propofol contains a total of 14 patients. Allergy to propofol was more likely in patients with a history of atopy.[3] Allergic reactions to propofol on first exposure are usually because of the isopropyl groups that may act as epitopes, and which are present in various medications. allergic reaction if reexposure occurred is usually because of the phenol molecule.[2] We present a case of a known food allergy that underwent surgery under general anesthesia and developed serious near fatal cardiac arrest following propofol injection and was resuscitated successfully.

## CASE REPORT

We present the case of a 4-year-old boy presented for elective adenotonsillectomy. His parents reported of a medical history of eczema and multiple allergies to foods, which include milk, sesame, strawberry, eggs, soya beans. He had only one attack of asthma, which responded to Ventolin nebulizer and did not require hospitalization. On examination, the boy was fit and healthy. Preoperatively anaesthesia issues were discussed with the parents in detail to obtain an informed consent. In the presence of the mother the patient was induced via sevoflurane and 100% O_2_ An i.v. line was established followed by administration of fentanyl 20 mcg, propofol 20 mg followed by tracheal intubation. Dexamethasone 4 mg and metoclopramide 4 mg were given i.v to reduce postoperative nausea and vomiting. Following induction of anaesthesia, rapid desaturation and severe bronchospasm with difficulty to manually ventilate the lungs occurred, followed by bradycardia, and severe hypotension. Call for help was initiated and cardiac massage was started. Intravenous boluses of adrenaline were administered (10 mcg at a time), also i.v fluids were infused and patient was ventilated manually on 100% O_2_.

Patient responded to resuscitation with complete relief of bronchospasm and return of normal blood pressure. According to records from the monitors, bradycardia lasted for 3 min, before responding to resuscitation, while desaturation lasted for 7 min. Patient started to breath spontaneously with good bilateral air entry with no wheezes. Few minutes later he started awakening, opened his eyes spontaneously, and therefore the trachea was extubated in OR following which he was taken to the recovery room. In the recovery room, the patient was breathing spontaneously on room air, maintained oxygen saturation of 93–98%, no wheezes with normal blood pressure. He was fully conscious and talking to his parents. No blood sample was taken for tryptase level, as it is not part of the protocol for allergic reaction in the hospital. The incident was documented in the medical file and discussed in detail with the parents; we tried answering their questions and provided them with contacts of the anesthetist for follow-up and future quires. The boy was referred to the pediatrician for follow-up with a plan to refer him to allergy clinic in specialized center.

## DISCUSSION

Incidence of allergic reaction during anaesthesia is in the range of 1:10,000 to 1:20,000. As for drugs involved in perioperative anaphylaxis, muscle relaxant represented 69.2% of the incidence, while hypnotic was 3.7%.[4]

Propofol is more likely than other drugs used for induction of anesthesia to cause an allergic reaction, and 1.2% of cases of perioperative anaphylactic shock were attributable to propofol.[2] Literature review revealed that it is the glycoprotein found in food that is generally implicated as the allergenic component, and patients clinically allergic to egg possess IgE and IgG antibodies to protein fractions in egg. Therefore, patients who are allergic to eggs are generally allergic to egg protein or albumin, not lecithin (the egg phosphatides which are present in the ‘Diprivan’ emulsion).[5]

Bassett *et al*., reported an adverse allergic reaction to propofol in a patient with egg hypersensitivity and suggested that a history of egg allergy may have to be considered prior to administration of propofol.[6] Propofol is known to decrease respiratory resistance and may prevent bronchospasm, but allergy to propofol is more likely in patients with a history of atopy. It can cause a nonimmunologic, nonspecific histamine release, and this is more likely in patients with atopy.

Our patient’s allergic history is rather complicated, as he has definite multiple food allergy, as well as history of eczema and asthma. Besides this was his first exposure to anaesthetic drugs. Although bronchoconstriction could be the only presenting sign of anaphylaxis, an asthmatic attack was also in our differential diagnosis, i.e., asthma due to allergy, which could be supported by the good and rapid recovery of the patient and continued until the time for discharge. Bradycardia could be due to the known physiologic reflex to hypoxia in pediatric patients.

In conclusion, despite current evidence which suggests that egg allergic patients are not more likely to develop anaphylaxis when exposed to propofol, our patient has multiple food allergy including soybeans and has history of eczema and asthma, which might increase the risk of allergy to propofol. Referring of these cases to allergy clinic is important for subsequent anesthetic exposure.
